# High contamination rates of shoes of veterinarians, veterinary support staff and veterinary students with *Clostridioides difficile* spores

**DOI:** 10.1111/tbed.14034

**Published:** 2021-02-21

**Authors:** Joanna Wojtacka, Beata Wysok, Aleksander Kocuvan, Maja Rupnik

**Affiliations:** ^1^ Department of Veterinary Public Health Faculty of Veterinary Medicine University of Warmia and Mazury in Olsztyn Olsztyn Poland; ^2^ Faculty of Medicine University of Maribor Maribor Slovenia; ^3^ National Laboratory of Health, Environment and Food in Maribor Maribor Slovenia

**Keywords:** *Clostridioides (Clostridium) difficile*, environment, footwear, One Health, transmission, veterinary clinic

## Abstract

*Clostridioides difficile* is often found in animals and their environment. However, not much has been reported on veterinary clinics environment in terms of the spore load, prevalence and PCR ribotype diversity. The aim of this study was to assess the prevalence of *C. difficile* on shoe soles of veterinarians, veterinary support staff and veterinary students at the Veterinary Faculty campus. Altogether, 50 shoe sole swabs were collected, and the positivity rates ranged from 86.7% in swabs from veterinarians to 100% in swabs from support staff and students. Non‐toxigenic and toxigenic strains representing toxinotypes 0, IV and XIX were isolated and distributed into 17 different PCR ribotypes, most common being 010, 014/020, SLO002 and 009. PCR ribotype 010 was the most prevalent and isolated from shoe soles sampled in 6/7 areas. Students' shoes had highest ribotype diversity (15/17 PCR ribotypes) but showed a low overlap with ribotype isolated from vets and support staff shoes. Veterinary students are likely the main vectors of *C. difficile* spores transmissions among veterinary teaching clinics and the hospital.

## 1 INTRODUCTION

*Clostridioides difficile* is apart from being the cause of infection in humans also an important animal pathogen (Rodriguez Diaz et al., 2018). It has been detected in both symptomatic and asymptomatic animals of diverse species including wild animals and birds (Andrés‐Lasheras et al., 2016; Jardine et al., 2013), but is best studied in farm animals and pets (Rabold et al. 2018; Rodriguez Diaz et al., 2018). Variety of PCR ribotypes were reported in animals, many of them are common also in humans and in environment (Álvarez‐Pérez et al., 2017; Janezic et al., 2012).

Regardless of their health condition, animals shed the spores to the environment and animal reservoir is one of the potential sources for human *C. difficile* infections (CDIs) in the community (Lim et al., 2019). Prevalence of *C. difficile* in animal farm environment is well documented (Bandelj et al., 2017; Hopman et al., 2011; O’Shaughnessy et al., 2019). The pathogen was isolated from the trucks transporting food animals (Álvarez‐Pérez et al., 2018), from the carcasses and intestinal contents (Candel‐Pérez et al., 2019) but also in the environment of the slaughterhouse (Wu et al., 2017). Fertilization with manure or compost contributes to contamination of the soil and water (Brown & Wilson, 2018). Dogs' paws were described as a possible source of *C. difficile* spores in households (Janezic et al., 2018). Moreover, dog's nasal discharge has been lately reported as a possible new source of *C. difficile* transmission (Rodriguez et al., 2019).

The veterinary clinics and hospitals are also likely to be contaminated, but only few studies report the presence of *C. difficile* spores in this environment (Rodriguez et al., 2016). The prevalence of *C. difficile* spores was estimated during the screening for different zoonotic pathogens in 101 veterinary hospitals in Canada (Murphy et al., 2010), in the Large Animal Clinic and Small Animal Clinic of the Ontario Veterinary College (Weese et al., 2000), and lately in Veterinary Hospital of Complutense University of Madrid (Villagómez‐Estrada et al., 2019) showing highest contamination loads for isolation areas (29%) and on telephone, keyboards and taps (15%), dog walk entry (100%) and floor (8%), respectively. None of the reports has focused on the role of veterinary team on carriage the spores on their shoes, a sampling site that has recently gained much attention. First and subsequent reports from the United States and Bangladesh (Alam et al., 2014, 2017; Islam et al., 2019) showed varying loads of *C. difficile* on shoe swabs among all samples taken in the households and other urban areas. High level of shoe sole contamination was noted not only in households but also in hospitals in Slovenia (Janezic et al., 2018, 2020).

The aim of this study was to assess the *C. difficile* contamination rates on the shoe soles at the Faculty of Veterinary Medicine that conducts multidirectional professional activity in the field of veterinary services including didactic activities for the students.

## 2 MATERIALS AND METHODS

### 2.1 Sampling

**FIGURE 1:**
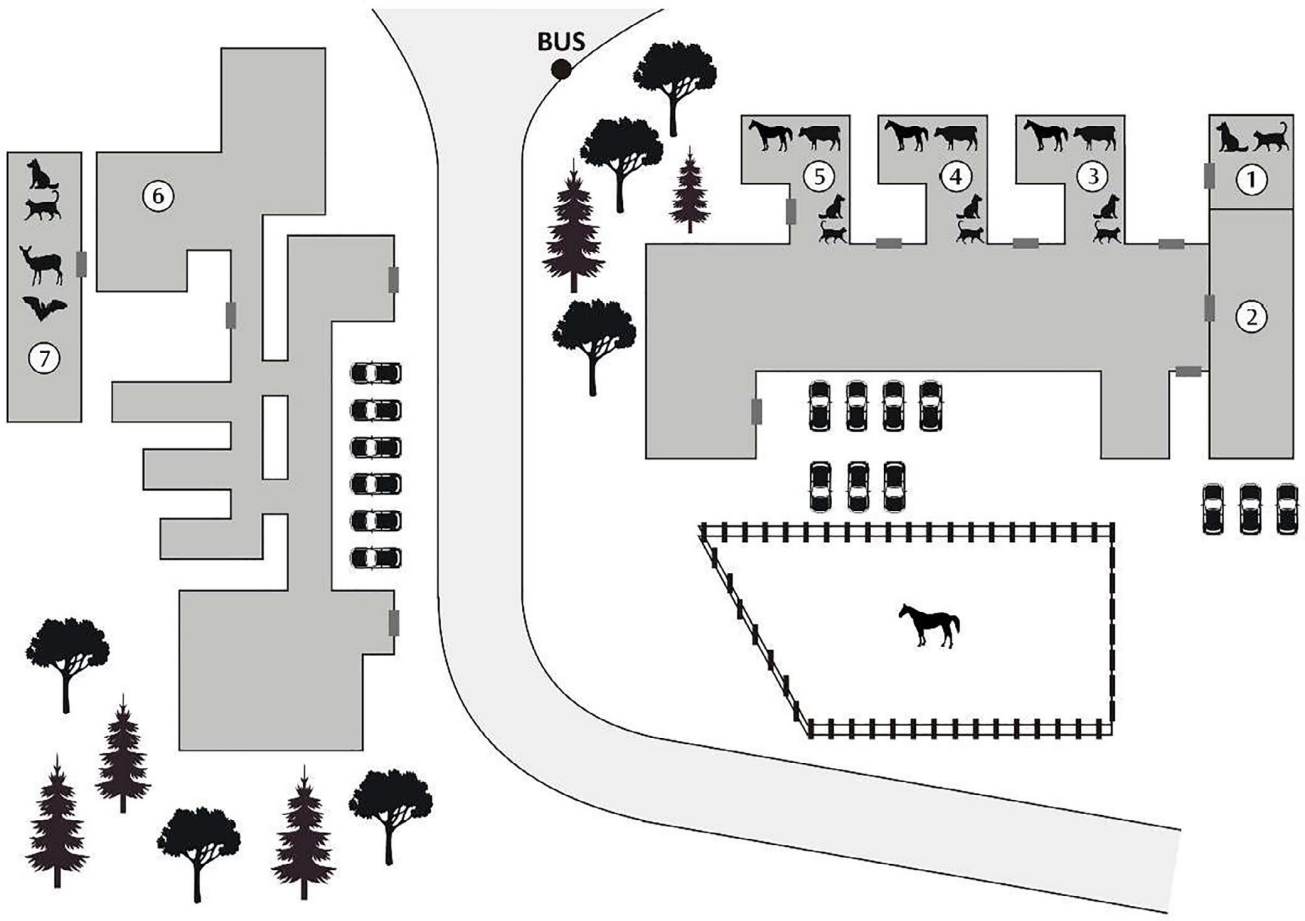
Schematic presentation of sampling locations. (1. Small Animal Clinic; 2. Food Hygiene Department; 3. Surgery Referral Clinic; 4. Internal Diseases Referral Clinic; 5. Reproduction Referral Clinic (including Ambulance crew); 6. Pathology Department; and 7. Infectious Diseases Referral Clinic)

The shoe sole swabs (*n* = 50) were collected from veterinarians (*n* = 15), support staff (*n* = 11) and veterinary students (*n* = 24) at one of the Faculties of Veterinary Medicine in Poland in February 2020. The samples were collected at seven different locations (Figure 1) from veterinarians and staff and in six out the seven locations also from students attending the classes. The sponges (3M™) pre‐moistened with NaCl (10 ml per sponge) were used for swabbing. After the sampling, the sponges were kept at 4°C until testing. A pair of shoes was swabbed with one sponge.

### 2.2 *Clostridioides difficile* cultivation

Cultivation was performed as described previously (Janezic et al., 2018). Each sponge was transferred to fresh sterile bag with 150 ml of BHI (Brain Heart Infusion, Biolife) medium supplemented with 0.1% l‐cysteine (Sigma‐Aldrich), 0.5% yeast extract (Biolife), 0.1% taurocholic acid sodium salt (Roth) and *C. difficile* selective supplement (SR0096E, Oxoid) and incubated in anaerobic conditions for 3–5 days. Subsequently, 1.0 ml of enrichment culture was subjected to ethanol shock (each sample in duplicate) by adding 1.0 ml of absolute ethanol for 30 min. After centrifugation at 10,000 rpm for 10 min, pellet was resuspended and inoculated on selective medium CHROMID^®^ *C. difficile* (BioMerieux) and incubated anaerobically for 3 days. After incubation, up to 6 suspected colonies were plated on Columbia agar with 5% horse blood (BioMerieux). Putative colonies of *C. difficile* were first screened based on the colony morphology and identified by mass spectrometry (MALDI‐TOF Biotyper System, Bruker).

### 2.3 PCR ribotyping

Crude DNA was isolated in a 5% (w/v) Chelex‐100 Resin (BioRad) solution. The isolates were first screened by analysis of PCR ribotype patterns on Agarose BioReagent (Sigma‐Aldrich). Only a single representative for clusters with identical profile and isolated from the same sponge was further PCR ribotyped on Certified Low Range Ultra Agarose (BioRad) after which the banding patterns of the ribotypes obtained were compared to those from in‐house library consisting on almost 300 PCR ribotype reference strains with the use of BioNumerics software v7.6 (Applied Maths, Belgium).

A single representative per PCR ribotype from each sample was stored at −80°C.

### 2.4 PCR toxinotyping

Toxinotypes were determined according to Rupnik et al. (1998) and Rupnik and Janezic (2016). Briefly, amplification and restriction of PCR fragment A3 (*tcd*A) and B1 (*tcd*B) were performed. Detection of *cdt*B was performed as described by Stubbs et al. (2000). Amplification of 115bp long insert with primer pair Lok1/Lok3 was performed to confirm non‐toxigenic strains (Braun et al., 1996).

### 2.5 Pulse‐field gel electrophoresis (PFGE)

PFGE was performed after restriction with *Sac*II as described by Janezic and Rupnik (2010). Standard protocol was modified, and the increased volume of proteinase K was used in the case of the strains that could not be typed (Fawley & Wilcox, 2002). The results were analysed with the use of BioNumerics software v7.6 (Applied Maths, Belgium).

## 3 RESULTS

**FIGURE 2:**
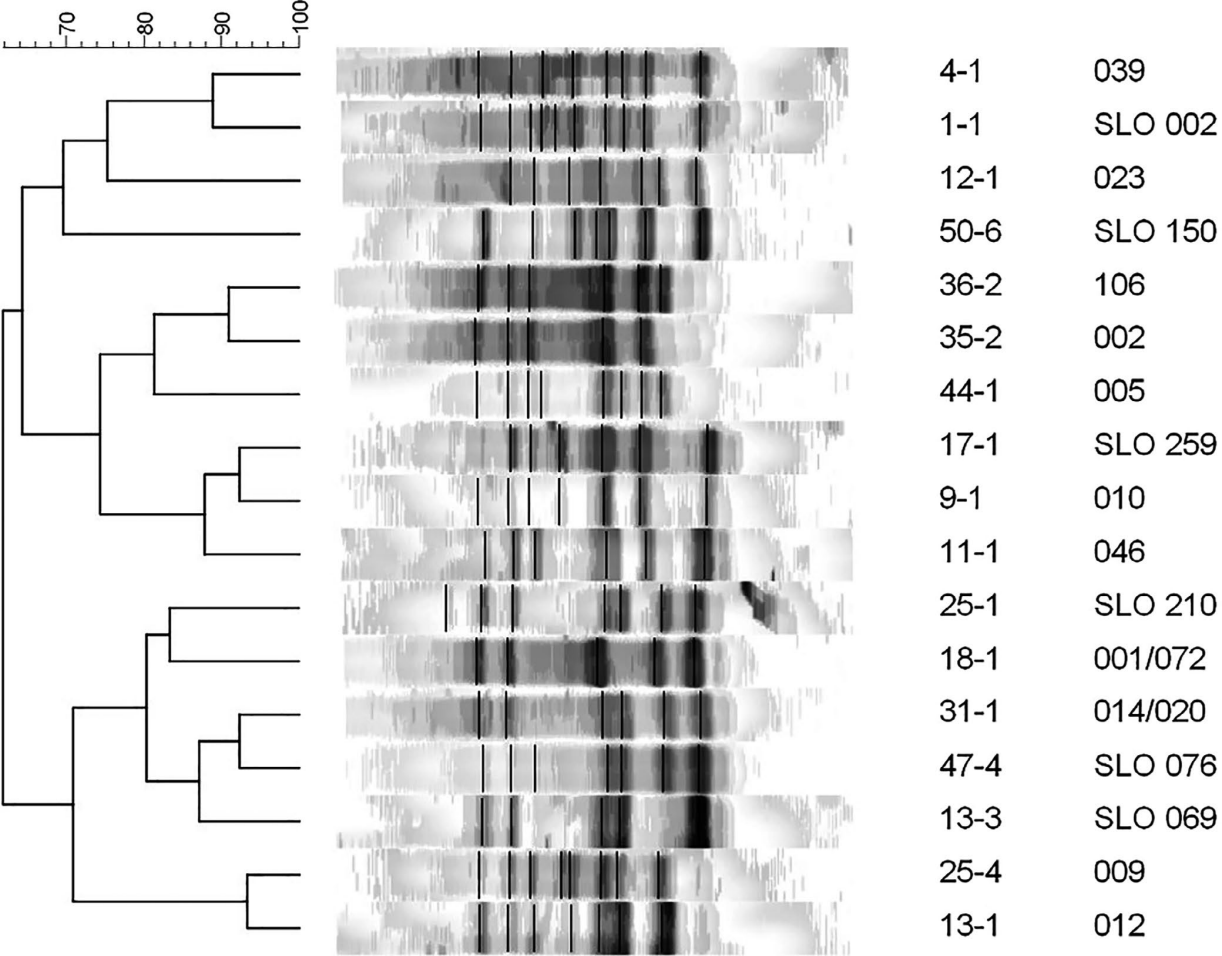
Representative strains of seventeen PCR ribotypes found among all *Clostridioides difficile* isolates obtained from shoe soles of veterinarians, supporting staff and students

Of 50 analysed shoe sole samples from different groups of volunteers (staff, veterinarians, students) from different departments at Veterinary Faculty, 48 (95%) were presumptively positive for *C. difficile* and 267 isolates were obtained. Ten of them represented other anaerobic spore‐forming bacteria, that is *Clostridium butyricum, Terrisporobacter glycolicus*, *Clostridium clostridioforme* and *Clostridium cadaveris*. The remaining 257 isolates were identified as *C. difficile* and distributed into 17 different ribotypes (Figure 2).

### 3.1 Prevalence of *Clostridioides difficile* and ribotype distribution in shoe soles across different volunteer groups

*Clostridioides difficile* was detected in the samples from all groups. In veterinary students, veterinary nurses and technicians all taken samples were positive. Two negative samples were obtained from veterinarians (Table 1) yielding the 86.7% positivity rate in this group.

**TABLE 1:** PCR ribotypes obtained from shoe soles of veterinarians, veterinary support staff and veterinary students

					Veterinarians	Support staff	Veterinary students
Tested samples (*n*)	na				15	11	24
*Clostridioides difficile*‐positive samples (*n*; %)	na				13 (86.7%)	11 (100%)	24 (100%)
PCR ribotype^a^	PCR ribotype toxinogenic status	Presence of toxin genes^b^	Number of samples per RT	Number of isolates per RT	Number of isolates	Number of isolates	Number of isolates
001/072	0	A^+^ B^+^ CDT^‐^	4	19	14	5	0
002	0	A^+^ B^+^ CDT^‐^	1	2	0	0	2
005	0	A^+^ B^+^ CDT^‐^	1	5	0	0	5
009	tox‐	A^‐^ B^‐^CDT^‐^	7	23	1	2	20
010	tox‐	A^‐^ B^‐^CDT^‐^	15	62	23	26	13
012	0	A^+^ B^+^ CDT^‐^	3	8	0	1	7
012	tox‐	A^‐^ B^‐^CDT^‐^	1	4	0	1	4
014/020	0	A^+^ B^+^ CDT^‐^	14	53	12	0	41
039	tox‐	A^‐^ B^‐^CDT^‐^	1	6	0	6	0
046	0	A^+^ B^+^ CDT^‐^	2	3	2	0	1
023	IV	A^+^ B^+^ CDT^+^	1	3	0	0	3
106	0	A^+^ B^+^ CDT^‐^	1	5	0	0	5
106	XIX	A^+^ B^+^ CDT^‐^	3	9	0	0	9
SLO002	tox‐	A^‐^ B^‐^CDT^‐^	8	38	9	24	5
SLO069	0	A^+^ B^+^ CDT^‐^	1	2	0	0	2
SLO076	0	A^+^ B^+^ CDT^‐^	1	1	0	0	1
SLO150	0	A^+^ B^+^ CDT^‐^	1	1	0	0	1
SLO210	0	A^+^ B^+^ CDT^‐^	1	4	0	0	4
SLO259	tox‐	A^‐^ B^‐^CDT^‐^	3	9	2	0	7
TOTAL			NA	257 (all strains)	63	64	130

Note

RT—PCR ribotype.

^a^ some PCR ribotypes included differed toxin gene profiles, and each of them is presented as a separate row.

^b^ A for gene *tcd*A, B for gene *tcd*B, CDT for gene *cdt*B; NA—not applicable.

In 20 samples, more than one PCR *C. difficile* ribotype was found; two different PCR ribotypes were found in 19 samples (14 students, 3 supporting staff, 2 veterinarians), and three different ribotypes were found in one sample (student) (Table 2).

**TABLE 2:** Multiple PRC ribotypes obtained from different sampling groups at different locations

Sample designation	Sample location	Volunteer group	PCR ribotypes isolated
25	Food hygiene department	Student	009, 010, SLO210
26		Student	001/072, SLO002
46		Student	009, 014/020
47		Student	014/020, SLO076
49		Student	009, 014/020
50		Support staff	014/020, SLO150
35	Pathology department	Student	002, 014/020
36		Student	010, 106
37		Student	010, 106
38		Student	014/020, 106
39		student	014/020, 106
40		Student	014/020, 046
13	Surgery referral clinic	Student	012, SLO069
14		Student	010, 012
15		Student	009, SLO259
18	Reproduction referral clinic	Veterinarian	001/072, 009
32		Student	012, 014/020
11	Small animal clinic	Veterinarian	SLO002, 046
51		Support staff	009, 010
33	Infectious diseases referral clinic	Support staff	SLO002, 012

PCR ribotypes with the highest number of isolates were 010, present on shoes from all analysed groups, and 014/020 present on shoes from vets and students (Table 1). The most prevalent PCR ribotypes found on the highest number of samples were 010, 014/020 and SLO002.

All groups shared only three of 17 PCR ribotypes. These were 009, 010 and SLO002. All PCR ribotypes isolated from veterinarian shoes were present on shoes from supporting staff and/or students. One PCR ribotype (039) was found only on the support staff shoes. The student shoes were the source of the majority of isolated ribotypes (88.9%) and consequently showed the highest diversity. Eight ribotypes (002, 005, 023, 106, SLO069, SLO076, SLO150 and SLO210) found on student shoes were not present neither on veterinarian shoes nor on the supporting staff shoes (Table 1).

### 3.2 Prevalence of *Clostridioides difficile* and ribotype distribution in different sampling areas

The samples were collected in seven different areas dealing with companion, farm and wild animals (Figure 1). There was a high PCR ribotype variability noted in terms of the sampling location (Table 3).

**TABLE 3:** PCR ribotypes obtained in different sampling areas

Sampling area	No of tested samples	No of *Clostridioides difficile*‐positive samples (%)	No of PCR ribotypes	PCR ribotypes
Small animal clinic	11	11 (100%)	6	009, 010, 039^a^, 046, SLO002, SLO259
Food hygiene department	11	10 (90,9%)	8	001/072, 009, 010, 014/020, SLO002, SLO150^a^, SLO210^a^, SLO076^a^
Surgery referral clinic	6	6 (100%)	6	009, 010, 012, 023^a^, SLO069^a^, SLO259
Internal diseases referral clinic	4	3 (75%)	2	010, SLO259
Reproduction referral clinic (including Ambulance crew)	9	9 (100%)	6	001/072, 005^a^, 009, 010, 012, 014/020
Pathology department	7	7 (100%)	5	002^a^, 010, 014/020, 046, 106^a^
Infectious diseases referral clinic	2	2 (100%)	3	012, 014/020, SLO002
Total numbers	50	48 (95%)	17 different ribotypes	

^a^ PCR ribotype present in only one sampling area.

Given the high overall positivity rate, it is not surprising that *C. difficile* was present in all tested locations. Two to eight PCR ribotypes were found at given location (Table 3). The only two negative samples were collected at Food Hygiene Department and at Internal Diseases Referral Clinic.

The PCR ribotype diversity at a specific location is in congruence with the number of samples taken (Table 3). The most prevalent PCR ribotype 010 was isolated from all sampling locations except from Infectious Diseases Referral Clinic. The next most prevalent PCR ribotypes 009 and 014/020 were each isolated from the samples taken in 4 different areas. PCR ribotypes 012, SLO002 and SLO259 were present at 3 locations, while 001/072 and 046 were found at two locations (Table 3).

The only binary toxin‐positive strain (PCR ribotype 023, toxinotype IV) was isolated from the student's shoes in the Surgery Referral Clinic.

### 3.3 Similarity between *Clostridioides difficile* strains across sampling sites and volunteer groups

**FIGURE 3:**
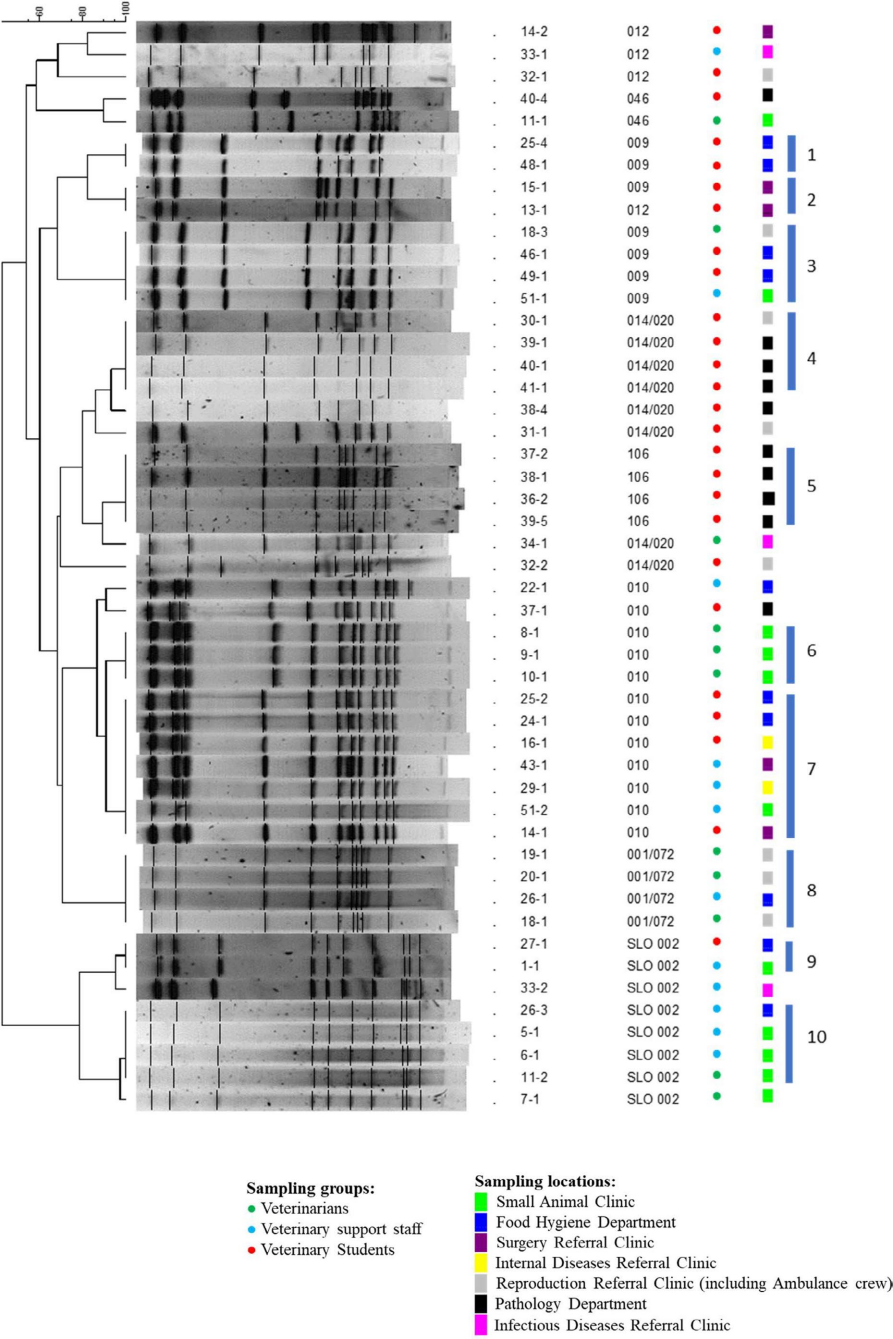
Pulse‐field gel electrophoreses profiles of *Clostridioides difficile* isolates from different sampling locations and different volunteer groups. Ten clusters of strains with identical PFGE profile were found indicated with vertical blue line

Selected strains from PCR ribotypes found in several locations, and/or different volunteer groups were typed by PFGE to assess their identity. The results show that isolates within a given ribotype cluster together but can have identical or diverse PFGE profiles (Figure 3). PCR ribotype 014/020 grouped together with ribotype 106. PCR ribotype 012 was distributed between two main branches; while three strains formed coherent (but non‐identical) group, a single strain showed identical PFGE profile as RT 009 strain. This RT 012 strain also had tox‐ profile, same as ribotype 009. The ribotyping and toxinotyping were repeated for this strain with the same results.

Few clusters of potentially identical strains were detected (Figure 3). Only one of them includes strains from two PCR ribotypes (009 and 012; cluster 2, described above). Each of the clusters 1, 5 and 6 includes strains isolated from different samples but in the same location and same volunteer group. All other clusters contain strains from two or more locations and two or more volunteer groups. The most numerous cluster of seven strains from PCR ribotype 010 (cluster 7) was spread across four locations and was found on student and support staff shoes.

## 4 DISCUSSION

Studies on *C. difficile* prevalence in the environment of veterinary settings are not numerous (Madewell et al., ,^1995^, ^1999^; Murphy et al., 2010; Rodriguez et al., 2016; Struble et al., 1994; Villagómez‐Estrada et al., 2019; Weese & Armstrong, 2008), and to date, only one of them included *C. difficile* on the footwear of the personnel (Weese et al., 2000). Their reported positivity rate for the footwear of medical personnel in veterinary clinics was 12.5%. This is substantially lower compared with our results showing 86.7% of veterinarians and 100% supporting staff carrying *C. difficile* spores on their shoes. In non‐hospital human reservoir, the reported rates are also lower and vary from 26.4% (Alam et al., 2017) through 39.7% (Alam et al. 2014) to 43% (Janezic et al., 2018). In contrast, in hospital environment the footwear positivity rate increases to 62%, but differed between two hospitals and between wards (Janezic et al., 2020). High positivity of shoe soles could imply the ongoing *C. difficile* outbreak, but the high PCR ribotype diversity did not confirm this. Similar as in hospitals also here in the veterinary setting, the overlap between ribotypes from different clinics/departments was low.

We have not sampled the animals upon arrival or the floors; therefore, we cannot speculate on the source of spore contamination. Floors were shown in other studies to be often contaminated by diverse *C. difficile* strains. Environmental *C. difficile* in veterinary teaching hospital was isolated from the floor surface by Weese et al. (2000). The contamination rates were 5.9% and 8% for Small and Large Animal Clinics, respectively. A report from Canada based on results obtained from 101 small animal clinics (Murphy et al., 2010) showed 16% positivity rate for the floors. Latest report from Spain shows that contamination rate for *C. difficile* in veterinary teaching hospital (floor) can be as low as 8% (Villagómez‐Estrada et al., 2019). In Spain, Villagómez‐Estrada et al. (2019) isolated 4 different ribotypes from the floor sampled in different areas of the veterinary teaching hospital, that is 078, 154, 014 and 039; two of which were found also in our study. However, our results show much higher diversity of PCR ribotypes disseminated on shoe soles from different clinics and areas in the Veterinary Faculty. Our isolates belonged to ribotypes well known from human, animal and environmental studies (Davies et al., 2016; Janezic et al., 2016; Rodriguez et al., 2019; Silva et al., 2015). Veterinarian and support staff shoes had notably lower numbers of different *C. difficile* ribotypes (7/17 and 6/17, respectively) when compared to student's shoes (15/17 different ribotypes). Clusters of identical strains from different sampling points indicate that shoes can contribute to spore transmission between the clinics. Whole‐genome sequencing (WGS) would further confirm the clonality of strains within the clusters. Although all three groups (veterinarians, veterinary staff and students) had comparable positivity rates, students are the ones who contributed the most to the diversity and several PCR ribotypes were found only on their shoes. Because of this difference in the diversity of PCR ribotypes between students and employees, we concluded that students are more likely to collect the spores on their shoes outside the clinics/departments. Thus, students could be considered as a significant source of contamination of the veterinary clinics and hospital from the external (outdoor) environment and vectors of the spore transmission between the departments and to other environments. However, the environmental samples within the clinics/departments or in the outdoor environment were not taken to experimentally support this assumption.

The shoes used in the environment of teaching veterinary clinics and hospital were highly contaminated with *C. difficile*. PCR ribotypes exhibit high variety and high overlap with common ribotypes found in humans, animals and environment. Several clusters of identical strains were found indicating transmissions within and between the areas. Changing shoes for the time spent in veterinary clinic/hospital practised by the employees seems to significantly reduce diversity of *C. difficile* ribotypes, probably suggesting also reduced transmission levels. The protocols for changing shoes while entering particular teaching units at Veterinary Faculties should be considered.

## CONFLICT OF INTEREST

None.

## AUTHOR CONTRIBUTIONS

JW contributed to study design, experimental work, data curation and manuscript drafting. BW contributed to sample collection. AK contributed to experimental work. MR contributed to study design, supervision and manuscript preparation. All authors have read and approved the final version of the manuscript.

## ETHICAL APPROVAL

Not applicable.

## Data Availability

Data available on request from the corresponding author.
